# Actin stabilization in cell migration

**DOI:** 10.3389/fcell.2022.931880

**Published:** 2022-08-11

**Authors:** Carsten Baltes, Divyendu Goud Thalla, Uli Kazmaier, Franziska Lautenschläger

**Affiliations:** ^1^ Experimental Physics, Saarland University, Saarbrücken, Germany; ^2^ Organic Chemistry, Saarland University, Saarbrücken, Germany; ^3^ Centre for Biophysics, Saarland University, Saarbrücken, Germany

**Keywords:** actin, migration, miuraenamide, nucleus, adhesion

## Abstract

Actin is a cytoskeletal filament involved in numerous biological tasks, such as providing cells a shape or generating and transmitting forces. Particularly important for these tasks is the ability of actin to grow and shrink. To study the role of actin in living cells this dynamic needs to be targeted. In the past, such alterations were performed by destabilizing actin. In contrast, we used the natural compound miuraenamide A in living retinal pigmented epithelial (RPE-1) cells to stabilize actin filaments and show that it decreases actin filament dynamics and elongates filament length. Cells treated with miuraenamide A increased their adhesive area and express more focal adhesion sites. These alterations result in a lower migration speed as well as a shift of nuclear position. We therefore postulate that miuraenamide A is a promising new tool to stabilize actin polymerization and study cellular behavior such as migration.

## Introduction

**FIGURE 1:**
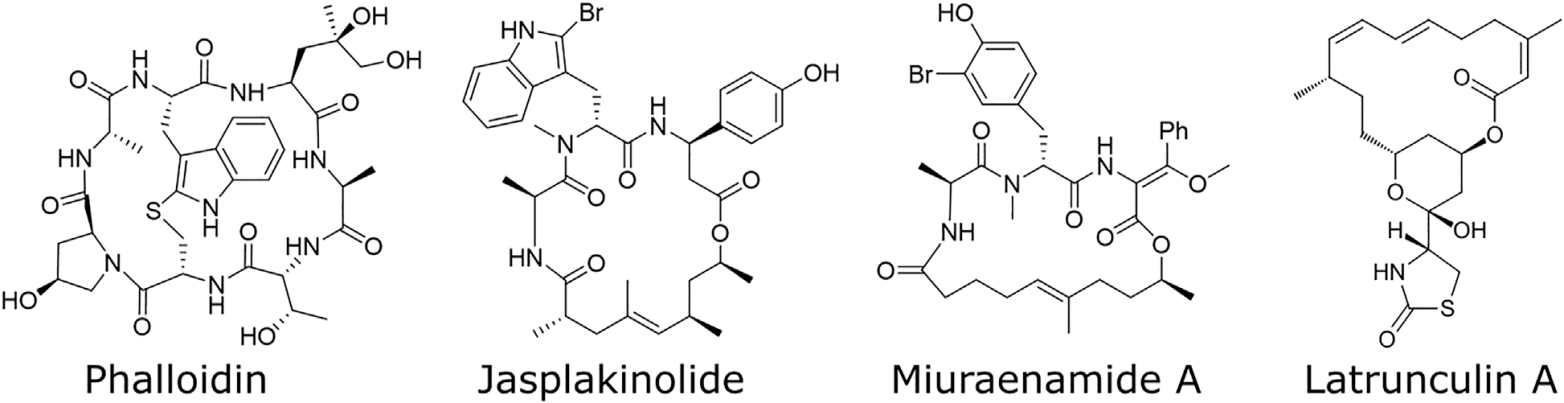
Structural formulas of various compounds binding to actin. Phalloidin, jasplakinolide, and miuraenamide A stabilize actin filaments, while latrunculin A destabilizes them.

Actin is one of the most preserved proteins in eukaryotic cells and is therefore involved in many cellular functions like cell division, migration, signaling and adhesion (Thomas and John, 2009). This variety of tasks illustrates its importance within living cells. Therefore, researchers are interested in understanding its role and its relevance by altering its properties and investigating the corresponding cellular behavior. To alter actin properties, actin binding compounds like phalloidin, latrunculin and jasplakinolide have been used (Figure 1). While actin depolymerizing compounds such as latrunculin have been part on many studies on the actin network, studying the effects of stabilized filaments remains challenging. The two most prominent compounds to stabilize actin filaments were phalloidin and jasplakinolide, both carrying major disadvantages: Phalloidin is not able to pass the cell membrane (Risinger and Du, 2020) which limits its use to fixed cells and the effect of jasplakinolide heavily relies on the used concentration and time scales (Ou et al., 2002). To bypass these disadvantages, we decided to use the alternative natural compound miuraenamide A (MiuA), which was isolated in 2006 from slightly halophilic marine myxobacterium (Iizuka et al., 2006). The structural relationship to jasplakinolide forced us to develop a total synthesis of MiuA (Wang et al., 2019) as well as other derivatives (Moser et al., 2017; Gegenfurtner et al., 2018) for structure-activity studies. By the synthetic protocols developed, miuraenamides are accessible on the gram scale for biological studies, e. g., regarding their possible binding mode (Wang et al., 2019), their effect on cell migration under chemotaxis (Moser et al., 2017) and their regulatory effects on gene expression (Gegenfurtner et al., 2018). Modifications to the structure of MiuA have also been shown to reverse the stabilizing properties of MiuA into a destabilizing compound (Wang et al., 2021), increasing its versatility.

In this study, we show the quantitative effects which MiuA has on the dynamics of actin filaments, as well as on the length of actin filaments in living cells. We additionally observed that treated cells occupied a larger area when allowed to freely spread and that their number of focal adhesions increased. We further found that MiuA treatment led to repositioning of the nucleus towards the cell center during migration and that cell migration speed decreased.

## Material and methods

### UV-patterning

We used two different types of patterns: “crossbow” patterns, that forced single cells to transform into a polarized shape and straight lines with a thickness of 10 µm to observe migration in 1D. For the production of the micropatterns, PEG-coated glass cover slips were placed on a photomask and illuminated with UV light according to the protocol of Azioune et al. (2010). We activated the photomask for 5 min before placing any glass objects on it and afterward put it back in for another 6 min. A fibronectin (concentration 25 μg/ml) solution (purchased from Thermo Fisher) was used to fill the holes among the PEG layer to create adhesive islands. For this procedure, the UV treated glass cover slips were placed upside down on a fibronectin droplet and kept either at room temperature for 1 h or placed in a sealed box inside a refrigerator (+ 4°C) overnight.

### Cell culture

RPE-1 cells transfected with LifeAct mCherry [as described by Maiuri et al. (2015)] and mouse embryonic fibroblasts (MEFs) were cultured at 37.5°C and 5% CO2 in Dulbecco’s Modified Eagle Medium Nutrient Mix F12 with 10% FBS, 1% GlutaMax and 1% Streptomycin + Penicillin (ThermoFisher). The RPE-1 cells were kindly given by the lab of Matthieu Piel, Institut Curie, Paris. The MEFs were kindly given by Dr. Jennifer Kasper, Leibniz Institut für neue Materialien, Saarbrücken.

### Miuraenamide A treatment

Miuraenamide A used in this study was obtained by total synthesis as reported previously (Karmann et al., 2015). It was given to cells 1 h prior to life cell imaging or fixation with PFA. Concentrations of MiuA were chosen to be 20 nM in each experiment. For this, MiuA has been added to the cell culture medium (DMEM/F12) which was given to cells and incubated for the duration of the experiments.

### Fixation of cells

Cell medium was removed, and cells were washed with PBS before adding a 4% PFA solution for 10 min. After that PFA was removed and samples were washed in PBS for 5 more minutes 3 times. Samples were then mounted with Fluoromount G + DAPI (Thermo Fisher) on a microscope slide, sealed with nail polish and stored at +4°C, protected from light.

### Paxillin staining

For visualization of focal adhesions, we took samples (RPE-1 LifeAct-mCherry) after the fixation with 4% PFA and dissolved the cell membrane. For this we used a 0.1% solution of TritonX-100 and put cells in it for 10 min. After three times washing with PBS we added a 3% BSA solution to them to block on specific binding for at least 1 h. A 1:1000 solution of paxillin antibodies (ThermoFisher, catalog nb. PA-34910) and 3%BSA was then added to the cells for another hour prior to washing with PBS and mounting the samples with Fluoromount G + Dapi on a microscope slide.

### Fluorescence microscopy

Fixed cells were imaged with a ZEISS Axio observer using a ×63 magnification oil objective. Life cell imaging was performed with a Nikon Eclipse Ti microscope using a ×10 magnification objective. Inside the microscope incubation chamber the temperature was set to 37°C and the CO_2_ concentration was set to 5%. The whole setup was allowed to stabilize at this temperature and CO_2_ concentration 1 h prior to the start of the experiments. Cell migration was observed by treating RPE-1 LifeAct mCherry cells with 250 ng/ml of Hoechst for 30 min before beginning the experiments and then taking pictures of them every 5 min.

### Actin staining for FRAP measurements and FRAP measurements

Dynamics of the actin network were measured by fluorescence recovery after photobleaching (FRAP) using a Zeiss LSM880 microscope. RPE-1 wild type cells were treated with BacMam2.0 (Thermo Fisher) at least 2 days before the experiment. The amount of BacMam used was set to 60 μL per 100,000 cells. Samples were placed in a glass bottom dish and were allowed to spread for at least 3 h before starting the FRAP measurements. Light with a wavelength of 405 nm was used to achieve the bleaching effects on single actin filaments. The parameters for the experiment were acquired using the protocol by Fritzsche and Charras (2015). Fluorescence intensity in areas of bleaching events was measured by the microscope software itself. A second and third ROI were set to measure the overall bleaching effect on the cell and the background signal. Final graphical presentation and statistical tests were performed using a home written Python3 script.

### MTT-assay

5,000 RPE-1 cells were placed inside several wells of a 96 well plate and the following five different conditions were chosen for testing:

• 1 µL DMSO per 1 ml medium, as (negative) control
MiuA (20 nM, 40 nM and 60 nM)
• 10 μg/mg mitomycin, as (positive) control

Cells were allowed to proliferate for 48 h under their respective conditions before the medium was removed and the cells were rinsed with PBS. MTT solvent at a concentration of 0.5 μg/ml (in cell culture medium) was added to the cells, before placing them in an incubator (37°C, 5% CO_2_) for 1 h. After the MTT solution was removed and the purple crystals that formed were dissolved in 100 µL of DMSO. To achieve a homogeneous dissolution, we placed the 96-well plate on a beacon shaker for at least 30 min. The light absorption, correlating with the number of cells inside each plate was then measured using a Tecan infinite 200 Pro, which automatically measures the absorbance coefficient in each well and provides xlsx files with the collected data. The machine was set to “multi-measurement” mode, meaning that nine distinct spots inside each well were measured and an average value for the absorbance coefficient was formed for each of them. The wavelength was set to 570 nm.

### Image analysis

All images were analyzed with Fiji (ImageJ) (Schindelin et al., 2012). Length of actin filaments was measured by hand using Fiji’s “line” tool. Time-lapse images of migrating cells were analyzed using the plug in “TrackMate” (Tinevez et al., 2017; Ershov et al., 2021). Nuclear distances were defined as the length between the back of the cell and the center of the nucleus divided by the total length of the cell. The number and size of focal adhesions were determined by paxillin staining. Paxillin signal was put under a threshold and then analyzed with Fiji’s build-in function “Analyze particles”, giving us the number and sizes of focal adhesions in those cells. All data were saved as csv. files and used for further analysis. Kymographs were performed using Fiji’s “Kymograph” tool.

### Statistical testing

Student’s t test were conducted on all experimental data and Pearson R values were calculated using a home build Python3 script. *p*-values were calculated and assigned as follows:

• p > 0.05: no significance (n.s.)
• p < 0.05: *
• p < 0.01: **
• p < 0.001: ***

## Results

### MTT assay

To confirm that a concentration of 20 nM MiuA is suitable for our experiments, we conducted a MTT assay on RPE-1 cells (Supplementary Figure S1). There we could see that cells treated with 20 nM of MiuA proliferated similar to the control group, while those treated with 40 nM MiuA showed the same behavior as the positive control group, treated with mitomycin C. When we increased the concentration further to 60 nM MiuA, we observed that the number of cells was even lower than that in our positive control group. Taking this into account, we decided to use 20 nM MiuA for all our experiments in this study.

### Actin dynamics

**FIGURE 2:**
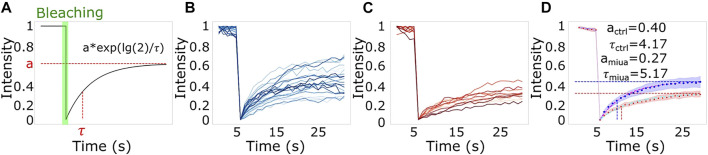
The effect of MiuA treatment on actin dynamics. **(A)** Scheme of a typical FRAP measurement and the used model to fit the data. FRAP measurements for **(B)** control and **(C)** MiuA treated cells. MiuA treatment increased the half time recovery and decreased the plateau value **(D)**. Cells were observed 5 s prior the bleaching. Number of cells: 20 (DMSO), 12 (MiuA).

To determine wether treatment with MiuA affected on the dynamics of actin filaments, we performed fluorescent recovery after photobleaching (FRAP) measurements. For this purpose, we bleached actin fibers in RPE-1 cells transfected with BacMan2.0 and measured the time evolution of the fluorescence intensity. We used BacMam staining as it stains G-actin and thus allows us to observe the network dynamics. Using a model for the recovery of the fluorescence intensity proposed by Fritzsche and Charras, (2015) we found that both the plateau level and the recovery time were altered in cells treated with MiuA. An alteration of the plateau level indicates a lower fraction of restored fluorescence and an alteration of the recovery time indicates a changing rate of exchanging actin monomers. Since upon treatment with MiuA, the plateau level decreased and the half-time recovery time increased (Figure 2), we concluded that MiuA treatment slows actin dynamics.

### Length of actin filaments

**FIGURE 3:**
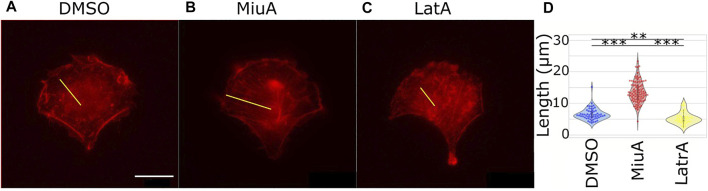
Length of actin filaments in geometrically identical RPE-1 LifeAct mCherry cells placed on a crossbow pattern. **(A)** Control cell (DMSO). **(B)** MiuA treated cell. **(C)** Latrunculin A treated cells. A yellow line indicates examples of how the length of actin filaments was measured. **(D)** Violinplots of the mean length of actin filaments in cells. Treatment with MiuA increases actin filament length, the treatment with latrunculin A decreases filament length. Number of cells: 58 (DMSO), 88 (MiuA), 38 (latrunculin A). Scale bar is 15 μm.

Because actin filament dynamics might influence the length of actin filaments, we next wanted to compare actin filament length depending on MiuA. Therefore, we first aimed to obtain geometrically identical cells so that we could compare similar structures (Théry, 2010). We placed RPE-1 cells that express LifeAct mCherry as a fluorescent dye on crossbow micropatterns (Figure 3). Once cells had a similar shape, compared the length of actin filaments in cells treated with 20 nM MiuA with the length of actin filaments in untreated cells. This concentration was chosen from the literature and was used throughout the study (Moser et al., 2017). Additionally, we used latrunculin A to destabilize the actin network as a negative control group. We manually analyzed the actin filament length using ImageJ. MiuA treatment resulted in a mean length of 13.57 µm compared to 6.26 µm in untreated cells and 4.89 µm in cells exposed to latrunculin A. Taken together, we can conclude that the length of actin filaments in MiuA treated cells increased significantly.

### Number of focal adhesions and spreading area

**FIGURE 4:**
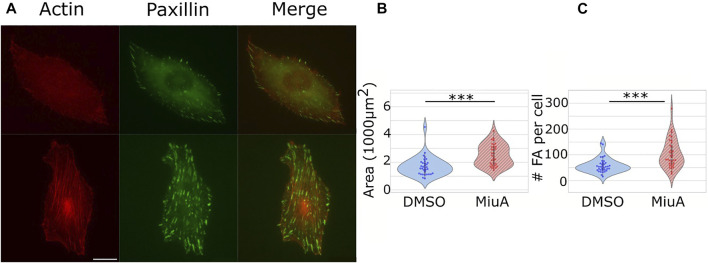
Effects of 20 nM MiuA on the spreading area and the number of focal adhesions in RPE-1 cells. **(A)** Top row: DMSO treated cells. Bottom row: MiuA treated cells. **(B)** The spreading area of MiuA treated cells increased significantly as well as the number **(C)** of focal adhesions. Number of cells: 35 (DMSO), 34 (MiuA). Scale bar is 15 μm.

We placed cells on micropatterns to compare cells which all had the same shape in order to compare the length of similar actin filaments. However, micropatterns are a rather artificial approach, which is helpful for understanding particular parameters, but is difficult to relate to the *in vivo* situation. Therefore, we next compared the adhesion of cells on 2D fibronectin coated glass surfaces. We quantified the number of focal adhesions in RPE-1 cells treated with MiuA: We stained for paxillin (Figure 4A), a protein involved in the formation of focal adhesions and counted the focal adhesions using ImageJ. Interestingly, the mean number of focal adhesions in MiuA treated cells increased from 57.829 in control cells to 103.235 in MiuA treated cells, resulting in an overall increase by a factor of 1.7 (Figure 4C). Following the number of focal adhesions, we also measured the spreading area of fully adhered cells. Consistent with the increased number of focal adhesions, MiuA treated cells occupied a significantly larger area than control cells. The spreading area increased by a factor of 1.5 from 1,693.06 µm^2^ in untreated cells to 2,605.78 µm^2^ in MiuA treated cells (Figure 4B).

### Migration behavior and position of nuclei

**FIGURE 5:**
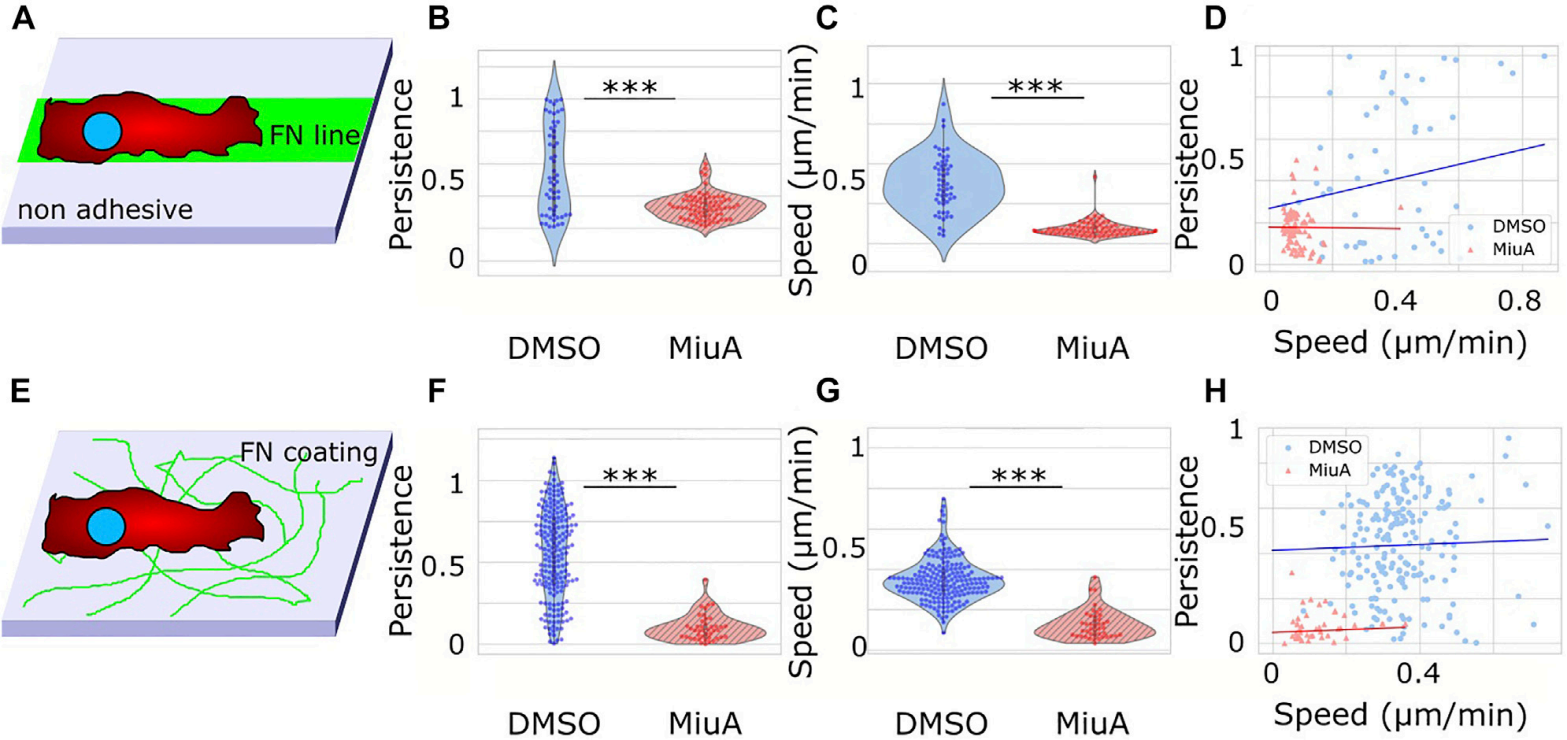
Migration of RPE-1 cells on fibronectin coated surfaces. **(A)** Scheme of the experimental setup for 1D migration on lines. **(B)**, **(C)** Violin plot of the persistence and speed of migrating cells. When treated with 20 nM MiuA, the persistence and speed decreased significantly. **(D)** Plotting the persistence of cells against their speed revealed a positive correlation in the control group and a slightly negative correlation in MiuA treated cells. Number of cells 1D: 55 (DMSO), 77 (MiuA). **(E)** Scheme of the experimental setup for 2D migration on a surface. **(F)**, **(G)** ) Violin plot of the persistence and speed of migrating cells. When treated with 20 nM MiuA, the persistence and speed decreased significantly. **(H)** Plotting the persistence of cells against their speed revealed a slightly positive correlation in both groups. Number of cells 2D: 197 (DMSO), 43 (MiuA).

To understand how elongated actin filaments, a higher number of focal adhesions and larger spreading areas affect the migration behavior of RPE-1 cells, we placed cells on fibronectin lines of 10 µm width as well as on a fibronectin coated glass surface and recorded their migration behavior (Figure 5). RPE-1 cells were chosen for their mesenchymal migration properties and their use in other migration studies (Maiuri et al., 2015; Terriac et al., 2019). We tracked cellular movements by staining the nuclei with Hoechst and taking pictures every 5 min. We analyzed the resulting trajectories using the ImageJ plug in TrackMate. Upon treatment with MiuA, cellular movement in 1D decreased significantly from 0.372 μm/min in the control case to 0.09 μm/min in the MiuA treated cells (Figure 5C) as well as the persistence of cellular movement, which decreased from 0.442 in untreated cells to 0.168 in cells exposed to MiuA (Figure 5B). Kymographs of those cells also showed a reduction in membrane activity during migration (Supplementary Figure S2). The same effect occured in cells migrating on a fibronectin coated glass surface. Their speed and persistence decreased after treatment with MiuA from 0.346 μm/min to 0.124 μm/min and from 0.444 to 0.079, respectively (Figures 5F,G). We also calculated the Pearson R correlation value between the speed and the persistence of migrating cells (Figures 5D,H), revealing an R value of 0.35 for untreated cells and −0.01 for MiuA treated cells in 1D. In the 2D case the R value stayed the same for both conditions at 0.06.

We also performed a 2D migration experiment using MEFs and observed the same effects (Supplementary Figure S3). The mean speed and persistence of MEF cells dropped from 0.259 μm/min and 0.311 to 0.125 μm/min and 0.153 respectively.

**FIGURE 6:**
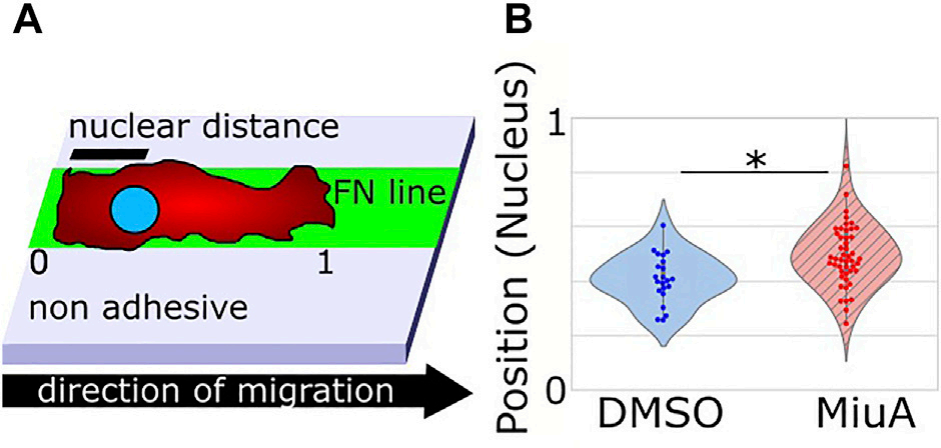
**(A)** Scheme of the experimental setup to determine the position of the nucleus. 0 and 1 refer to the back end and the front end of the cell respectively, while nuclear distance means the distance between the back end of the cell and the nucleus. **(B)** Violin plot of the positions of the nucleus in migrating cells. When treated with 20 nM MiuA, the nucleus was shifted toward the cell center.

Additionally, we measured the position of the nuclei in RPE-1 cells during migration on fibronectin lines. We imaged the nuclei by Hoechst staining and analyzed the position within the cell using ImageJ. Interestingly, the position of the nuclei of cells treated with MiuA significantly shifted toward the cell center (Figure 6B).

## Discussion

Actin, which is omnipresent in eukaryotic cells, has various in living systems (Thomas and John, 2009). Therefore, altering aspects of actin always affects many aspects of the entire system, increasing the challenge of understanding single actions of actin in cells such as the role of actin filament length in cell migration, proliferation etc. One way to study actin is by compounds which stabilize or destabilize actin by altering the polymerization rates. Although both types of such compounds are well known since the end of the 20th century, research has mainly focused on actin destabilizing compounds like latrunculin A or cytochalasin D (Risinger and Du, 2020). Nevertheless, it is not sufficient to only destabilize actin, but means of stabilizing actin are needed. However, stabilizing actin filaments remains challenging, as the two most prominent compounds, phalloidin and jasplakinolide, have serious disadvantages. Phalloidin cannot pass through cell membrane, rendering it impossible to use this compound in living cells. The second compound, jasplakinolide, stabilizes actin filaments in living cells. However, this stabilizing effect relies on the concentration and the duration of treatment (Ou et al., 2002). Therefore, handling jasplakinolide is challenging and often not reproducible. In our study we used a synthetic sample of the natural compound miuraenamide A, a secondary metabolite of a halophilic myxobacterium isolated from soil samples of the Japanese coast (Iizuka et al., 2006). This marine actin stabilizer (Ojika et al., 2008) can, similar to jasplakinolide, pass through the cell membrane and is therefore suitable for observing living cells. Due to its structural similarity to other cyclodepsipeptides, MiuA also targets actin filaments (Iizuka et al., 2006; Ojika et al., 2008; Sumiya et al., 2011; Karmann et al., 2015; Ojima et al., 2016). We therefore used MiuA to test the effect of actin stabilization on the dynamic behavior of actin inside living cells. The overall dynamics of actin filaments were decreased by treatment with MiuA. We showed that the half time recovery of actin filaments in MiuA treated cells increased in FRAP measurements meaning that the dynamics of the filaments decreased. These data are supported by the results of Florian A. Gegenfurtner and colleagues showing that the diffusion of actin monomers in the cytoplasm of MiuA treated cells is reduced when compared to the control group (Gegenfurtner et al., 2018). Following the change in actin dynamics due to MiuA, we wanted to see how the architecture of actin filaments in living cells might be affected. In 2019, Shuaijun Wang and colleagues showed that actin filaments *in vitro* exposed to MiuA increased their elongation rate and overall length, as well as the number of filaments (Wang et al., 2019). This finding matches our observations in living RPE-1 cells, where treatment with MiuA induces longer actin filaments. Because these data were taken on micropatterns, we then moved to RPE-1 cells on 2D and compared their adhesion capacity regarding number of focal adhesions and spreading area. Christina Moser et al. observed no significant change in the spreading area of HUVECs treated with MiuA after 90 min of spreading time (Moser et al., 2017). This finding is in contrast to our study, where we found that RPE-1 cells exposed to MiuA occupy a larger area than the control group. One explanation for such differences might be the different time scales used in our experiments. As we seeded the cells on fibronectin coated glass surfaces, we allowed the cells to fully adhere for at least 4 h. We then treated them with MiuA for 1 h prior to fixing the cells with 4% PFA. The longer adhesion time might explain the significant difference in the spreading area of MiuA treated cells compared to the work of Moser et al. We also counted the number of focal adhesions per cell and found a significantly increased number. Nevertheless, the interplay between the spreading area and the number of focal adhesions remains open for further studies. Adhesion and actin are directly linked to migration. One study using MiuA investigated 2D chemotaxis in HUVECs treated with MiuA and showed no change in migration speed (Moser et al., 2017; Wang et al., 2019). However, in our study, RPE-1 and MEF cells treated with MiuA showed a significant decrease in mean speed compared to the control group. This difference between both studies might be because we investigated 1D and 2D migration on fibronectin without chemotaxis. Regarding other possibilities of altering the migration of cells, Ali et al. (2021) showed that jasplakinolide affects the phosphorylation of alpha-1-syntrophin, which in turn leads to a decrease in motility. As MiuA and jasplakinolide have a similar molecular structure (Karmann et al., 2015), MiuA might also be capable of interfering with the alpha-1-syntophin pathway. We also reproduced the correlation between speed and persistence that have been shown by Maiuri et al. (2015). Thus fast cells in 1D migrate in a more persistent manner. The treatment with MiuA, as well as the migration on 2D surfaces in general resulted in low migration speed paired with R values close to zero, meaning we could draw no conclusion about the correlation between these two values. As actin plays an active role in the positioning of the nucleus during cell migration (Thomas and John, 2009; Gardel et al., 2010; Calero-Cuenca et al., 2018), we measured the position of the nuclei in the migrating cells while being on fibronectin lines and under treatment with MiuA. Because disassembly of actin filaments is a crucial step during mesenchymal migration (Louise, 1999) and the organization of the actin network is linked to the position of cell organelles (Gardel et al., 2010) we hypothesized that the position of the nucleus during migration might also be affected by a change in the actin network. Indeed, we measured a repositioning of the nucleus toward the cell center under MiuA treatment. Our data are supported by the finding that the treatment of fibroblasts with jasplakinolide resulted in an increase in both cell body movement and in lamellipodia (Louise, 1999). As MiuA and jasplakinolide show similar effects on the actin cytoskeleton and their molecular structures are related (Karmann et al., 2015), we assume that MiuA might also affect the mechanism responsible for positioning the nucleus. However, further investigations are needed as the mechanism itself and its link to the cytoskeleton were not revealed by our study.

In this work we show that miuraenamide A is a powerful tool to affect the dynamics and architecture of the actin cytoskeleton. Following this finding, we showed that actin filaments play a crucial role in the positioning of nuclei in migrating cells, as treatment with MiuA induced not only longer filaments but also shifted the nucleus toward the cell center. Furthermore, we could see that longer filaments lead to cells occupying a larger area and increasing their number of focal adhesions. In the future, we and others will be able to use this tool to further understand the role of actin in living cells.

## Data Availability

The raw data supporting the conclusions of this article will be made available by the authors, without undue reservation.
