# Supplementary material for: Actin stabilization in cell migration
Source: Front Cell Dev Biol. 2022 Aug 11;10:931880. doi: 10.3389/fcell.2022.931880 (PMC9403840; doi:10.3389/fcell.2022.931880)
Supplement: Supplementary file 1 [file DataSheet1.pdf]

Suplementary figures

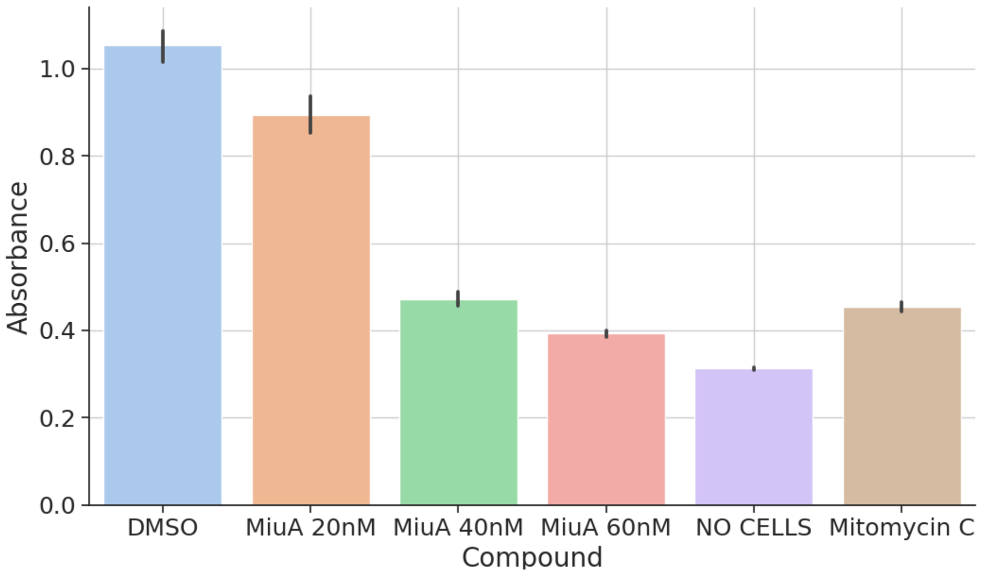

SI Figure 1: MTT assay for the viability of RPE-1 cells treated with MiuA. When the concentration of MiuA reached 40 nM, the number of living cells was equal to the number of cells treated with a cell cycle stopper (Mitomycin C). When the concentration of MiuA reached 60 nM, the number of living cells decreased further. Errors bars showing the confidence interval of 95%. Three independent experiments were performed.

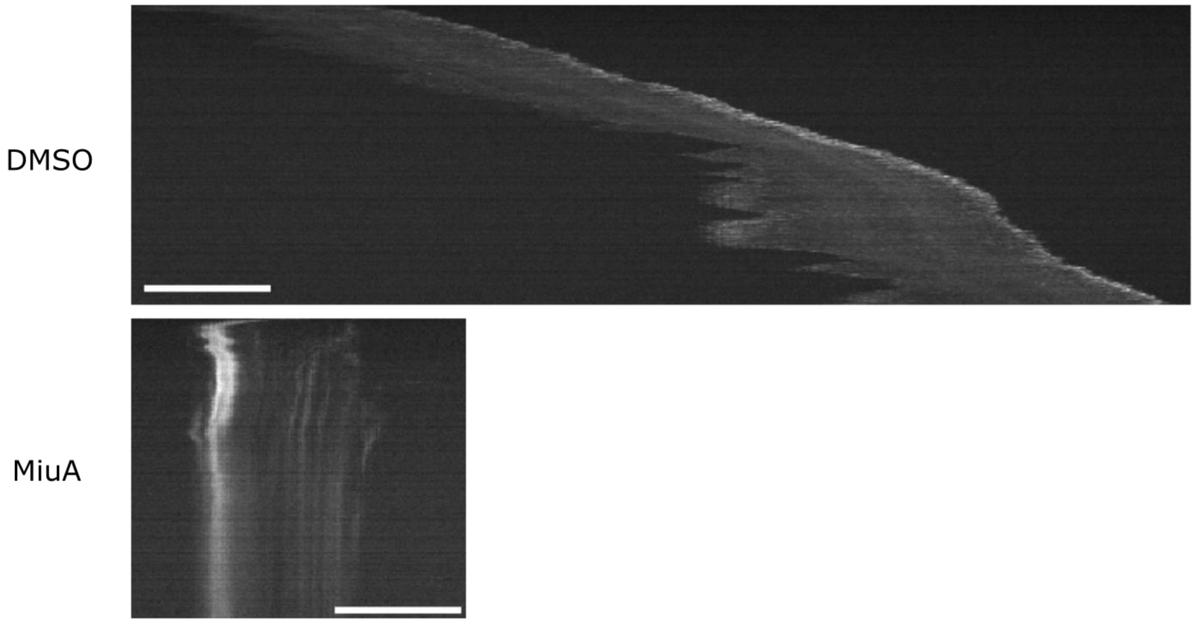

SI Figure 2: Kymograph of the movement of RPE-1 cells on fibronectin lines. Top: DMSO, bottom: MiuA treated cells. Scale bar is 15 µm

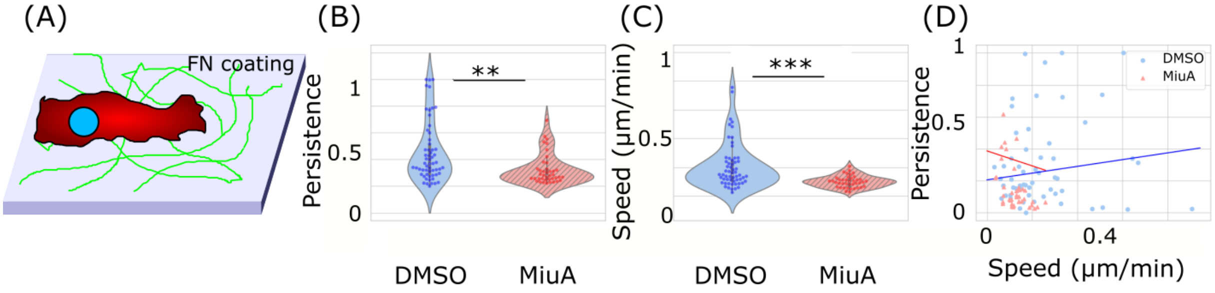

SI Figure 3 (A) Scheme of the experimental setup for 2D migration on a surface (MEFs). (B), (C) Violin plot of the persistence and speed of migrating MEFs. When treated with 20 nM MiuA, the persistence and speed decreased significantly. (D) Plotting the persistence of cells against their speed. Number of MEFs 2D: 58(DMSO), 40(MiuA).
